# Does the multiple sclerosis (MS) map need to change again? An update of MS prevalence in Mazandaran province of Iran in 2018

**DOI:** 10.1186/s12883-020-1618-6

**Published:** 2020-02-12

**Authors:** Seyed Mohammad Baghbanian, Hamed Cheraghmakani, Reza HabibiSaravi, Arash Azar, Fariba Ghasemihamedani

**Affiliations:** 1 Multiple Sclerosis Fellowship, Neurology Department, Boualicina Hospital, Mazandaran University of Medical Sciences, Sari, Iran; 2 Neurology Department, Boualicina Hospital, Mazandaran University of Medical Sciences, Sari, Iran; 3 Multiple Sclerosis Clinic, Boualicina Hospital, Mazandaran University of Medical Sciences, Sari, Iran; 4 School of Public health, Faculty of Medicine, University of Sydney, Sydney, Australia; 5 Iran University of Medical Sciences, Tehran, Iran

**Keywords:** Multiple sclerosis, Prevalence, Map, Mazandaran, Iran

## Abstract

**Background:**

Information of Previous studies on the prevalence of MS, including our study conducted 12 years ago, used to shape global prevalence map of MS. According to those results, Iran placed in medium-prevalence MS region in the world Atlas of MS 2013.This study aimed to investigate the prevalence of MS in Mazandaran province after 12 years and the need for possible changes in the global map of the prevalence of MS.

**Methods:**

We included all MS patients living in Mazandaran province in 2018 in this descriptive cross-sectional study. We updated our pre-existing registration questionnaires which included demographic information and medical data of MS patients by interview. We obtained the demographic profile of Mazandaran province from the most recent census in 2016 and the National Civil Registry of the Mazandaran province for calculating prevalence of MS.

**Results:**

The total number of MS patients in Mazandaran was 2418 (25.8% male and 74.2% female) with a female to male ratio of 2.9. Based on the local population of 3,332,556, (50.4% male and 49.6% female), this study showed a prevalence of 72.5 per 100,000 for MS in this region. The prevalence of this disease by gender was 37.1 per 100,000 for men and 108.5 per 100,000 for women. The mean (SD) age of the patients at the time of the study was 38.5 (10.1) years with a minimum of 15 and a maximum of 75 years. The most common type of MS was Relapsing-Remitting MS with 86%.

**Conclusions:**

All recent studies showed significant upward trend in the prevalence of MS around the world. Based on the results of our study and many other studies in Iran, the Atlas of MS prevalence map needs to be update. Iran’s status should be changed to the high-prevalence of MS in the new Atlas. Due to the increasing prevalence of MS, we suggest an adjustment in the Global MS Prevalence Scale.

## Background

The Multiple Sclerosis (MS), as a chronic disease of the Central Nervous System with a potentially progressive course, is one of the leading non-traumatic disabling diseases in young adults. Despite its relatively low prevalence, MS is accompanied by ponderous socio-economic effects on communities [1].

Even though some MS patients experience minimal disabilities during their lifetimes, a majority of about 60% of other MS patients are not without problems within 20 years after the onset of the disease. They experience a variety of complications with significant impacts on their quality of life and MS has financial burdens on society as well [2].

Despite the tremendous advances in medical science, there is no definite treatment for MS, and existing therapies either reduce symptoms or slow down the progression of the disease by weakening or modulating the patients’ immune system, leading to a disease with a chronic and long-standing course [3]. MS has a significant impact on patients and is associated with a wide range of complications which require appropriate treatment and facilities. Therefore, it is important to have accurate and up-to-date epidemiologic information for effective planning [4].

**Fig. 1:**
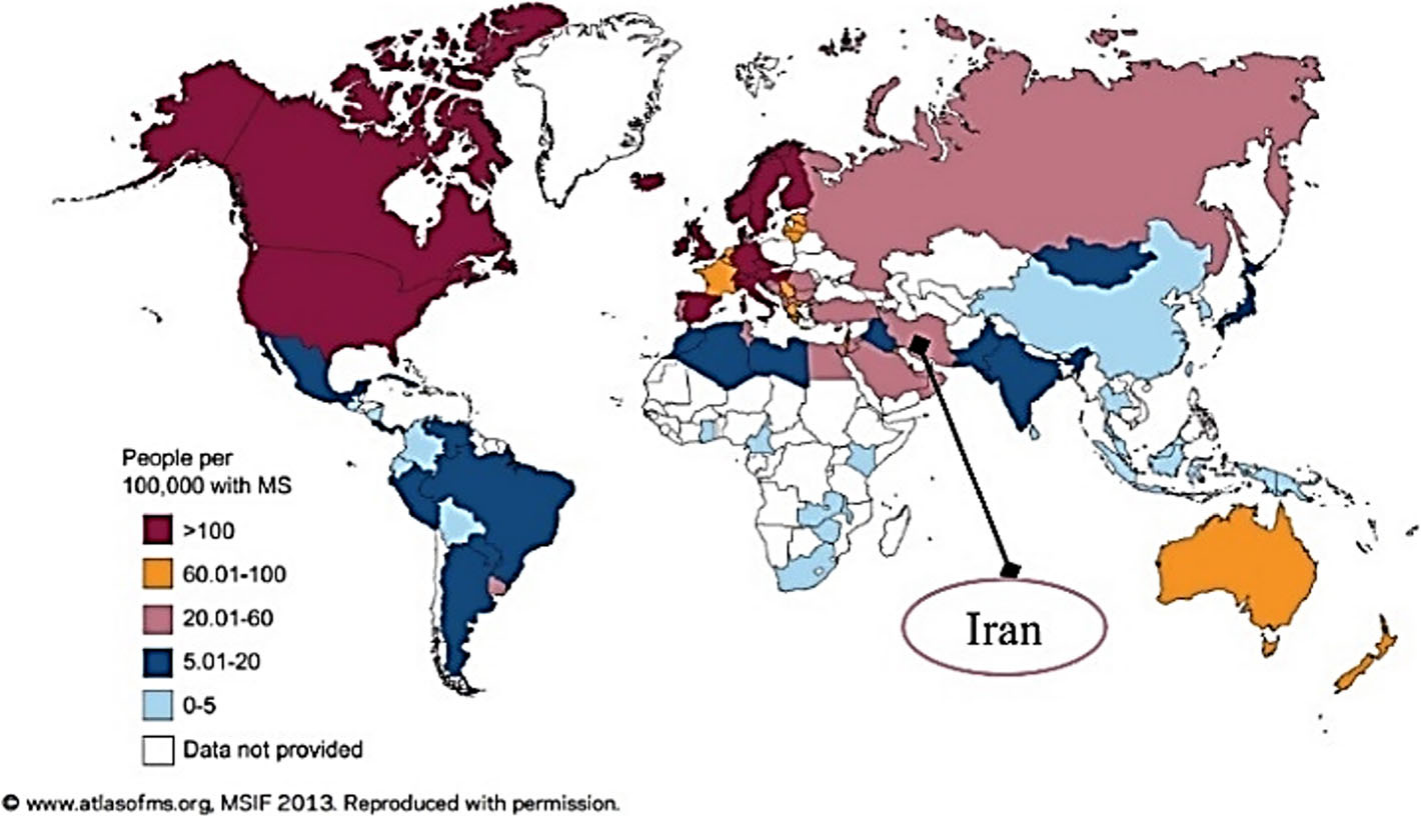
Map of MS prevalence in the world in 2013 [2]

Our previous study on the epidemiology of MS in Mazandaran province of Iran in 2007 showed interesting results about its prevalence and other epidemiologic indices in this province [4]. According to Kurtzke [5], Iran has placed in low-MS region in Atlas of the MS 2008 map. The information from our previous study, along with several other studies in the country [6], led to a change in Iran’s position in the MS prevalence map in the World Atlas of MS 2013 to the medium-prevalence category with 20–60 MS patients in 100,000 population [7]. (Fig. 1).

MS, as a chronic disease, is expected to have an increasing prevalence. As it was twelve years since the latest study on the epidemiology of MS in Mazandaran, health policy makers and directors needed updated information in this field for MS health planning. This study aims to assess the prevalence of MS in Mazandaran to update the information for policy making and health planning, and to provide a basis for future research projects in this field. The present study seeks an answer to this question: “Is there a need for a change in the prevalence map of MS in the world for the second time?”

### Study design

In this descriptive-analytical study, trained research assistants updated the demographic and medical information of MS registered patients in the MS Society of Mazandaran province using the standard registration questionnaire of the MS Society during Face-to-face or telephone interviews with the patients.

### Study population

The target population included patients with a confirmed diagnosis of MS who live in Mazandaran Province of Iran.

To be eligible for this study, patients needed to meet all the inclusion criteria and none of the exclusion criteria below at the time of registration for the study.

### Inclusion criteria

1. MS patients registered at the MS Society of Mazandaran to the end of 2018
2. Capability to provide consent

### Exclusion criteria

1. MS patients who were not residents of Mazandaran province
2. MS patients who were not willing to sign the consent form for this study

### Study method

During the process of registration of patients in the MS society of Mazandaran province, patients were assessed at a specialised committee of neurologists and were registered with a diagnosis of MS based on the 2018 McDonald criteria [8]. All the patients had completed their questionnaire of the MS Society at the time of registration. This questionnaire contained demographic (18 items) and disease status questions (12 questions).

This project registered and approved at the ethics committee of Mazandaran University of Medical Sciences, Sari, Iran (Protocol identification number: IR.MAZUMS.REC.1398.4778–4/20/2019). Informed consent obtained from all patients or their parents/guardians for those who were minors (age < 18) at the time of registration in the study for participation and disclosure of their information without mentioning their names in the research papers. We obtained the written or verbal (if illiterate) consent from all participants or their parents/guardians for those who were minors (age < 18) at the time of registration in their native language (Farsi). This method was approved by ethics committee of Mazandaran University of Medical Sciences, Sari, Iran. When participants were not capable of signing, written consent was obtained by fingerprint. The ability to provide consent was an eligibility criterion in this study. All participants were deemed capable of providing consent by their primary attendance in Mazandaran Multiple Sclerosis Society. Patients were granted their right to withdraw from the study at any time and for any reason.

After the registration for the study, a team of trained research assistants updated the pre-existing data with face-to-face or telephone interviews with patients, and another team of research assistant used this data to complete the electronic case report forms of our database.

We used the demographic data of Mazandaran obtained from the latest results of the 2016 census available at the website of Statistical Center of Iran [9]. Also, we obtained the estimation of the population of 2018 from the instant population statistics and forecast of the year 2018, available at the website of Mazandaran Civil Registry Office [10] to calculate the prevalence of MS.

After data entry, the study biostatistician used SPSS version 17 to perform analytical tests and to present the results as tables and charts. The Kolmogorov-Smirnov test was implemented to test the normal distribution of the data. When we needed hypothesis testing, in cases where the data had a normal distribution, the student t-test was used and the Kruskal–Wallis test was performed when the data set did not follow a normal distribution pattern.

## Results

**Fig. 2:**
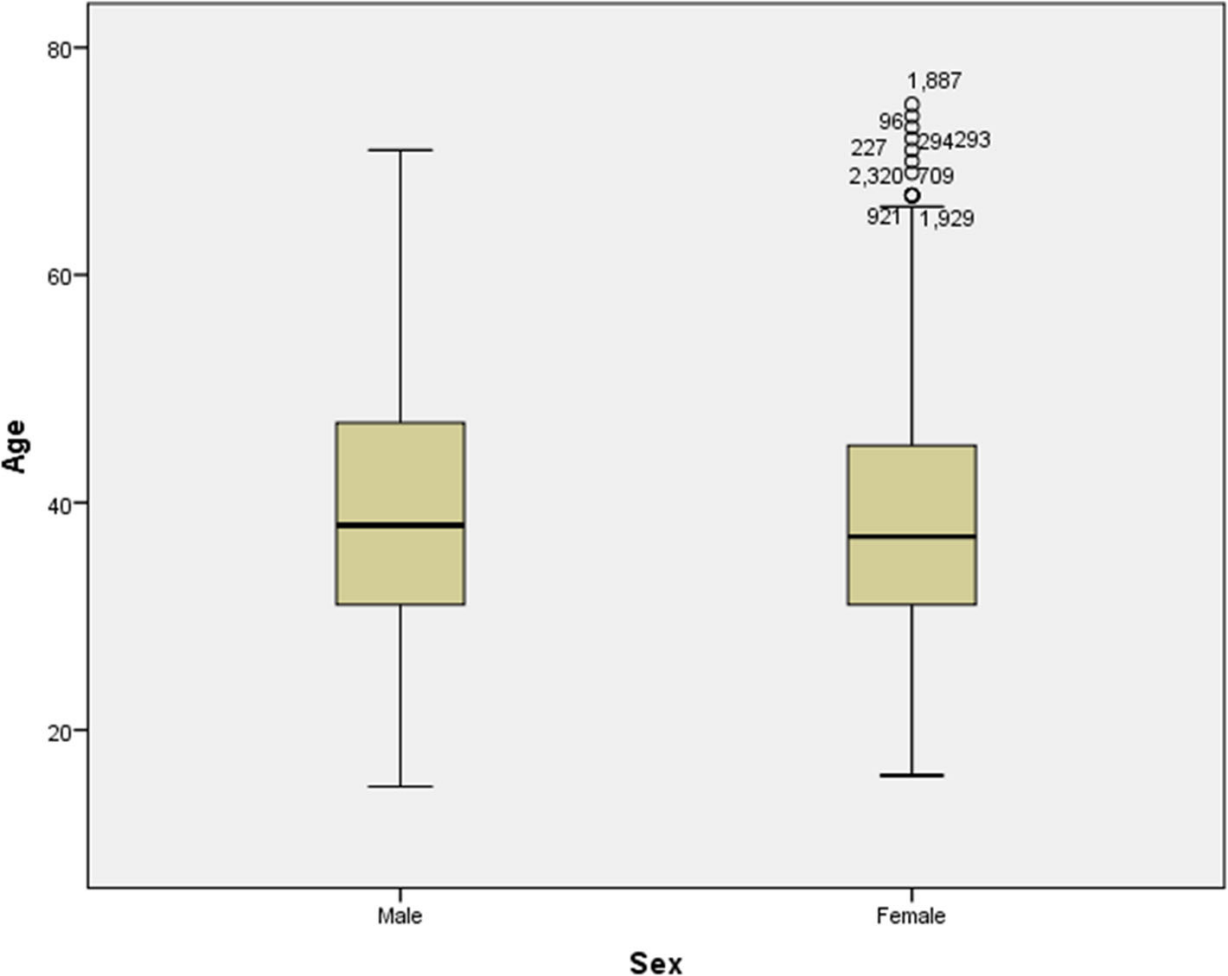
Distribution of MS patients by sex

The population of Mazandaran province at the time of the study was 3,332,556, of which 1,678,194 (50.4%) were male, and 1,654,362 (49.6%) were female. The total number of patients was 2418, of which 623 (25.8%) were male, and 1795 (74.2%) were female, leading to female to male ratio of 2.9. The prevalence of MS disease was 72.5 in one hundred thousand population. The prevalence of the disease by gender was 37.1 in men and 108.5 in women per one hundred thousand. The mean (SD) age of the patients at the time of the study was 38.5 (10.1 years) with a minimum of 15 and a maximum of 75 years. The mean (SD) age of patients was 39.2 (10.5) in men and 38.3 (9.9) years in women. (Fig. 2).

Regarding the marital status, married patients were the largest group with 1667 people (68.9%) while widows or widowers with 30 people (1.2%) was the smallest group. A large proportion of patients, (61%) had literacy levels of High school diploma to bachelor’s degree, and a significant per cent (29.8%) had an education level of primary school to high school diploma. The birthplace of 42% of the patients were rural areas from which 50% moved to urban areas subsequently. Only 301 (12.4%) of MS patients mentioned a history of immigration in their lives, with (62.1%) of them immigrating after the age of 15 years old. The complete demographic data of patients are shown in Table 1.

**Table 1:** Demographic Information of MS Patients in Mazandaran Province in 2018

		Male		Female		Total (percent) row	
		No.	%	No.	%	No.	%
Patients with MS in Mazandaran Province		623	25.8%	1795	74.2%	2418	100%
Marital status	Single	198	8.2%	442	18.3%	640	26.5%
	Married	412	17%	1255	51.9%	1667	68.9%
	Widow or Widower	1	^a^0.0%	29	1.2%	30	1.2%
	Divorced	12	0.5%	69	2.9%	81	3.4%
Education level	Illiterate	14	0.6%	92	3.8%	106	4.4%
	Elementary or lower than diploma	218	9%	503	20.8%	721	29.8%
	Diploma	152	6.3%	515	21.3%	667	27.6%
	Undergraduate	198	8.2%	610	25.2%	808	33.4%
	Master’s degree or higher	41	1.7%	75	3.1%	116	4.8%
Immigration history	Before 15 years old	25	8.3%	89	0.3%	114	37.8%
	15 years old or older	39	13%	148	49.2%	187	62.2%
Birth place	City	339	14%	1070	44.3%	1409	58.2
	Village	284	11.7%	725	30%	1009	41.7%
Current residence	City	433	17.9%	1299	53.7%	1732	71.6%
	Village	190	7.9%	496	20.5%	686	28.4%

^a^Because of displaying numbers up to one-tenth of decimal places, the numerical value obtained is zero

**Fig. 3:**
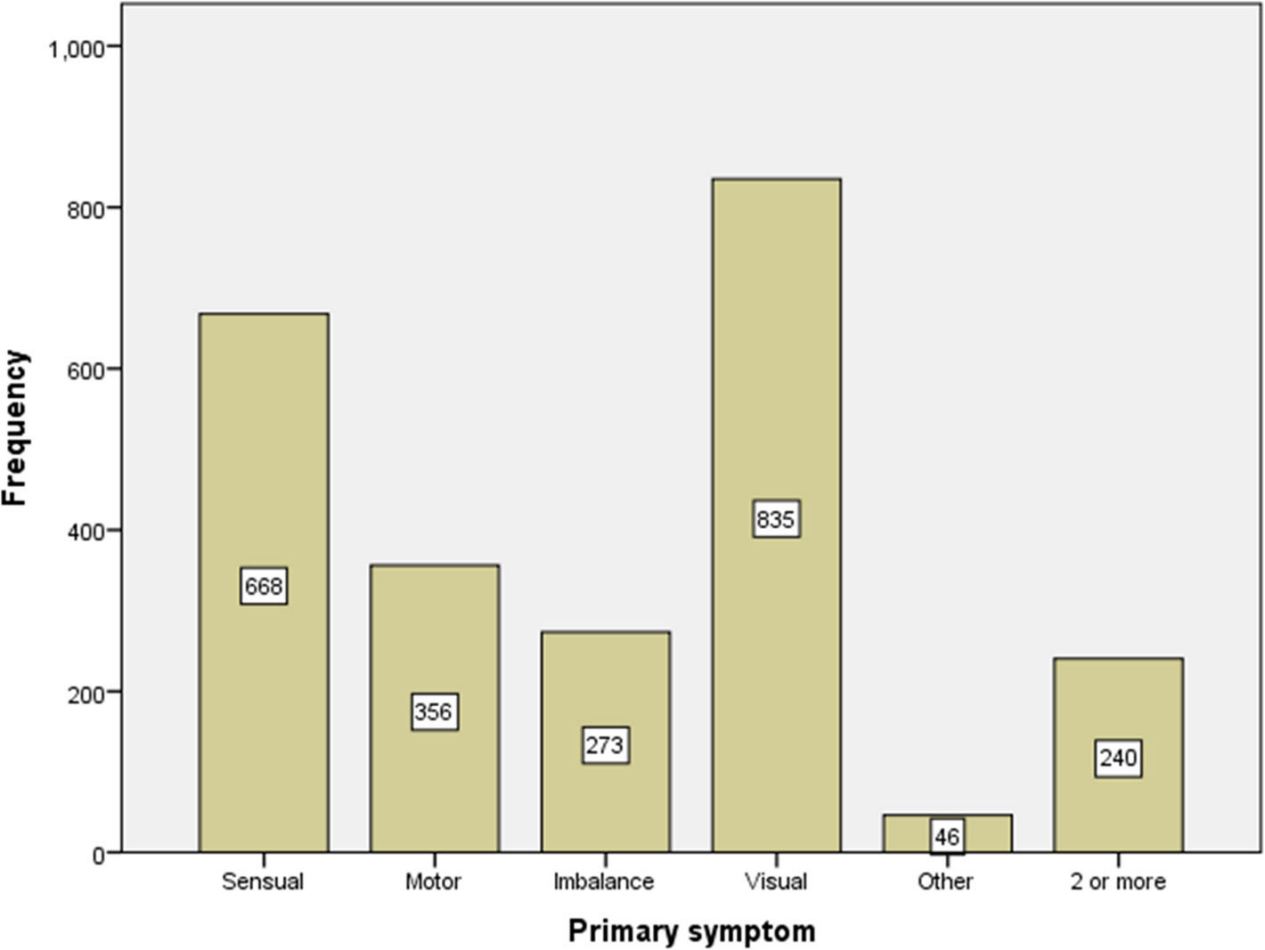
Frequency of symptoms at the onset of MS

Of the total number of patients, 2079 (86%) had a relapsing-remitting type of MS, and the progressive-relapsing type with 3 cases (0.1%) had the lowest number. The mean age (SD) of the initial symptom presentation was 28.7 (8.6) years, with a minimum of 6 and a maximum of 63 years. The most common symptom was visual disturbances (34.5%) and sensory problems (27.6%), and nearly 10% of the patients had 2 or more symptoms. (Fig. 3).

**Fig. 4:**
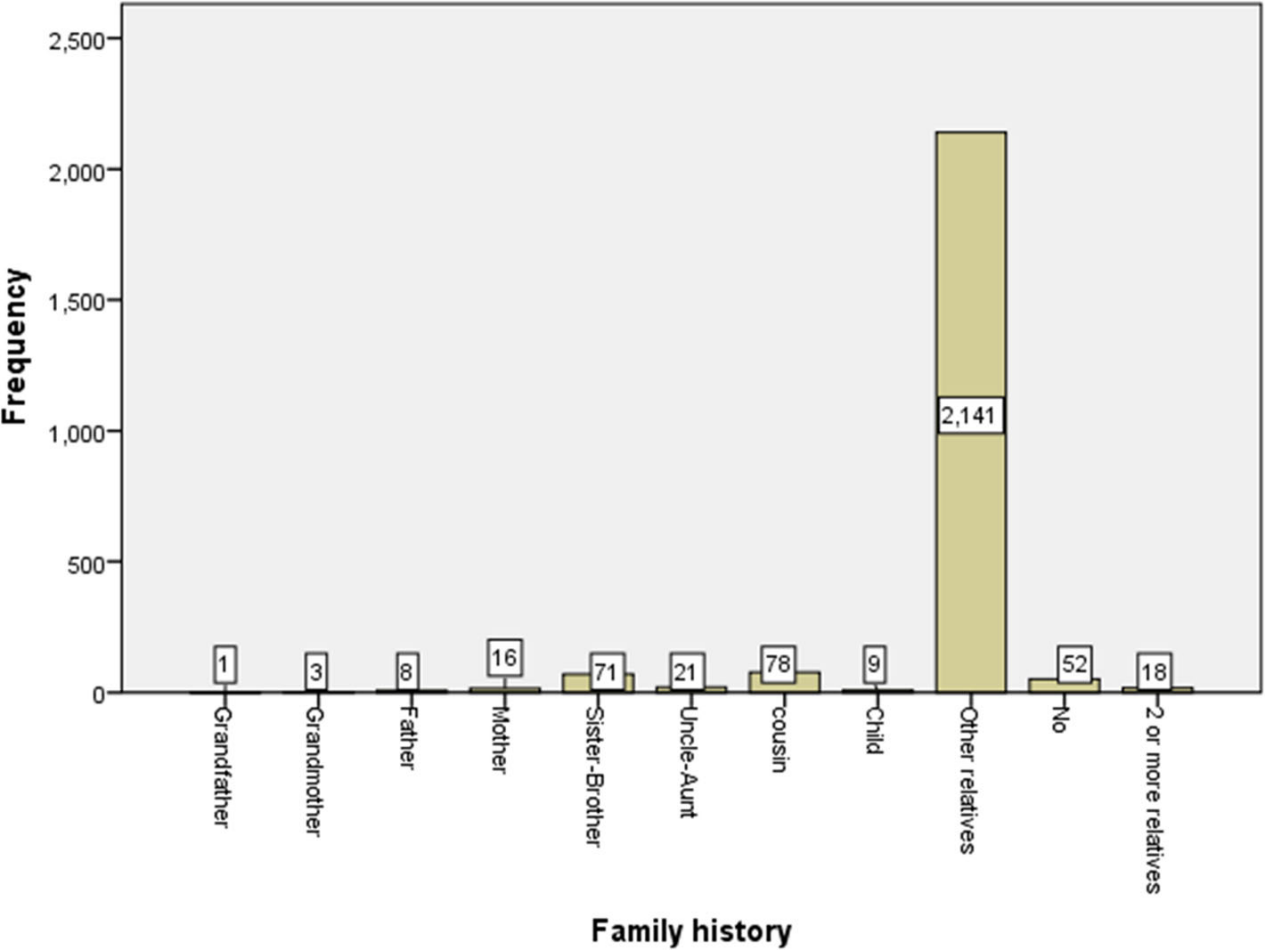
Family history of MS patients

Average elapsed time for reaching a final diagnosis was 15 days with a maximum of 20 years and a minimum of 3 days, and the difference in the time elapsed from the initiation of symptoms to the diagnosis of MS was not significant between men and women. Approximately 60% of patients reported having one or two hospital admissions only due to MS, while nearly one-third of the patients (33.5%) had no history of hospital admission, because they received out of hospital medical services. Most of the patients (88.5%) mentioned the history of MS in a non-close relative while only 52 patients (2.2%) had no family history of MS. (Fig. 4).

1941(80.3%) patients did not have any other health conditions and, rheumatologic diseases with a rate of 11.3%, were the most commonly associated co-morbidities. That was significantly greater in women (*p* < 0.05). The history of motor vehicle accidents before the occurrence of MS was 8.4, and 41.2% of patients had a history of surgery, while only a few patients (110(4.5%)) had blood transfusion or blood products usage history before the diagnosis of MS.

## Discussion

Over the past 10 years, there have been many epidemiological studies on MS in Iran indicating an increase in the prevalence of MS. Initial studies took place in Isfahan and Mazandaran provinces. The prevalence of MS was 20.1 per 100,000 people in Mazandaran Province (582 cases) in 2007. The average age of patients was 34.3 years, and the female to male ratio was 2.6 [4]. In the present study, it can be seen that the prevalence of MS in Mazandaran is 72.5 per 100,000 people, which shows an increase of roughly 3.5 times. The average age of the patients increased 4 years and reached to 38.5, and the ratio of women to men, by 0.3 increase, estimated at 2.9.

In Isfahan province which is located in central Iran, the prevalence of MS disease was 35.5 per 100,000 in 2006, and the average age of the patients was 32.5 years with a female to male ratio of 3.6 [6]. A more recent study in 2014 showed a 1.5-fold increase in the prevalence of this disease with 54.5 MS patients per 100,000 and a drop in the sex ratio of 0.3 since 2006 in the same region [11]. There was no MS patient under 15 years old. These findings suggest potential role of puberty period on the onset of pathogenesis of MS that noted in another study as well [12].

In 2009, a research project on MS showed a prevalence of 50.57 in 100,000 people for this disease in Tehran. The female to male ratio was 3.1 l, and the mean age of MS patients was 35.48 years in this study [13]. Izadi found the prevalence of 72.1 in 100,000 people in Fars province in 2015 for this disease with female to male ratio of 4.04 (a prevalence of 116.5 in women and 28.3 in men) [14]. A review article, published in “the International Journal of Epidemiologic Research” in 2018, showed a sharp increase in the prevalence of MS up to 95 per one hundred thousand since 2013 [15].

Our study showed a lower prevalence for MS in Mazandaran compared to other provinces of Iran with a lower latitude while, we expected Mazandaran, as a northern province, to have a higher prevalence of MS. Different factors can be contributing to a relatively lower estimation of the prevalence of MS in Mazandaran despite its higher latitude. One possible explanation for this difference can be the referral pattern of patients of neighbouring provinces to the study centres to access tertiary healthcare facilities. Because of the proximity of Mazandaran province to Tehran, the capital of Iran, with a significantly higher number of health care facilities, we can see a tendency in Mazandaran MS patients to follow their treatment in Tehran. Additionally, Rasht, the capital of Gilan province, might be more accessible for some of the patients in the western regions of Mazandaran.

Numerous studies have reported a similar increasing trend in the prevalence of MS in many other countries around the world. In 2002, Pugliatti et al. estimated the prevalence of MS in northern and western Europe to be under 170 per 100,000 population [16]. This figure increased to more than 200 in one hundred thousand in Kingwell et al. study in 2013 [17].. In Canada, there has been a dramatic increase in the prevalence of MS from 157 in 1996 to 265 per hundred thousand population in 2013 [18]. In the United States, the MS prevalence rate was estimated at 85 per 100.000 people in 2002 [19], while, 8 years later, studies conducted in three different regions of the United States, showed an increase of up to 109 per 100,000 population in the prevalence of this disease [20].

Although these findings hypothesized the sunlight and UV role in the pathogenesis of MS, but many studies did not support this hypothesis. In the study of Ofer Amram and et al., the life time UVB exposure estimated by new satellite technology [21]. There was no significant associatiation between the age MS onset and calculated UVB exposure [22].

## Conclusions

Repeated studies over the years have indicated a significant upward trend in the prevalence of MS throughout the world.

Concerning the current prevalence of MS in Mazandaran province along with other parts of Iran, changes in MS World Map will be expected. In the new release of the MS World Map, Iran is expected to be placed in the high-prevalence area category. Moreover, recent changes in the prevalence of MS in the rest of the world may result in further changes in this map.

Significant increase in the prevalence of MS in the World, suggests a new grouping in the Global MS Prevalence Scale. We recommend to categories countries by prevalence as follows: lower than 5 in 100,000 as an area of Very-low-prevalence, 5 to 20 in 100,000 as a low-prevalence-area, 20 to 100 in 100,000 as a medium-prevalence area, 100 to 180 in 100,000 as an area of high prevalence and above 180 in 100,000 as a very-high-prevalence area.

Recently introduced more effective medications has resulted in reduced or controlled progression of MS and prolongation of the duration of this disease. This increase in the life expectancy of MS patients leads to the increased cumulative prevalence of MS.

## Data Availability

The datasets used and analysed during the current study are available from the corresponding author on reasonable request.

## Abbreviations

MS: Multiple Sclerosis
